# Rapid-deployment aortic valve replacement during challenging aortic valve reoperation: a case report

**DOI:** 10.1093/jscr/rjad614

**Published:** 2023-10-31

**Authors:** Ari Mennander, Aino Saranko, Mika Kohonen

**Affiliations:** Tampere University Heart Hospital and Tampere University Medical School, Tampere, Finland; Tampere University Heart Hospital and Tampere University Medical School, Tampere, Finland; Tampere University Heart Hospital and Tampere University Medical School, Tampere, Finland

**Keywords:** rapid-deployment aortic valve replacement, reoperation

## Abstract

A previously implanted stenotic aortic valve bioprosthesis with stenotic coronary ostia and intramyocardial calcium was surgically debrided resulting in disruption of the left outflow track. A rapid-deployment aortic valve bioprosthesis was implanted to cover the remnant aortic valve annulus, ensure open coronary ostia, and secure a well-functioning aortic valve bioprosthesis with low postoperative gradient.

## Introduction

Aortic valve reoperation due to restenosis and recalcification of the outflow track after a previously implanted aortic valve prosthesis poses a surgical challenge. Previously, aortic valve reoperation has successfully been performed after a stenotic conduit prosthesis using a rapid-deployment aortic valve prosthesis [1–3] and a sutureless aortic valve prosthesis [4], but without injury to the left outflow track or aortic valve annulus. We describe our recent case of reoperation in a 67-year-old patient with stenotic coronary ostia adherent to the previously implanted stenotic aortic valve prosthesis using a rapid-deployment Edwards Intuity bioprosthesis (Edwards Lifesciences, Irvine, CA, USA) after disruption of the aortic valve annulus upon surgical revision of the calcified left outflow tract.

## Case report

The patient was a 67-year-old smoking man with hypertension and clopidogrel treatment due to a history of transient ischemic attacks.

Aortic valve replacement with a 25 mm size biological Trifecta prosthesis (Abbot, Abbot Park, IL, USA) and a supracoronary replacement of the ascending aorta using an interposition aortic Dacron prosthesis were performed 7 years earlier due to significant stenosis of a bicuspid aortic valve and a dilatation of the ascending aorta.

**Figure 1:**
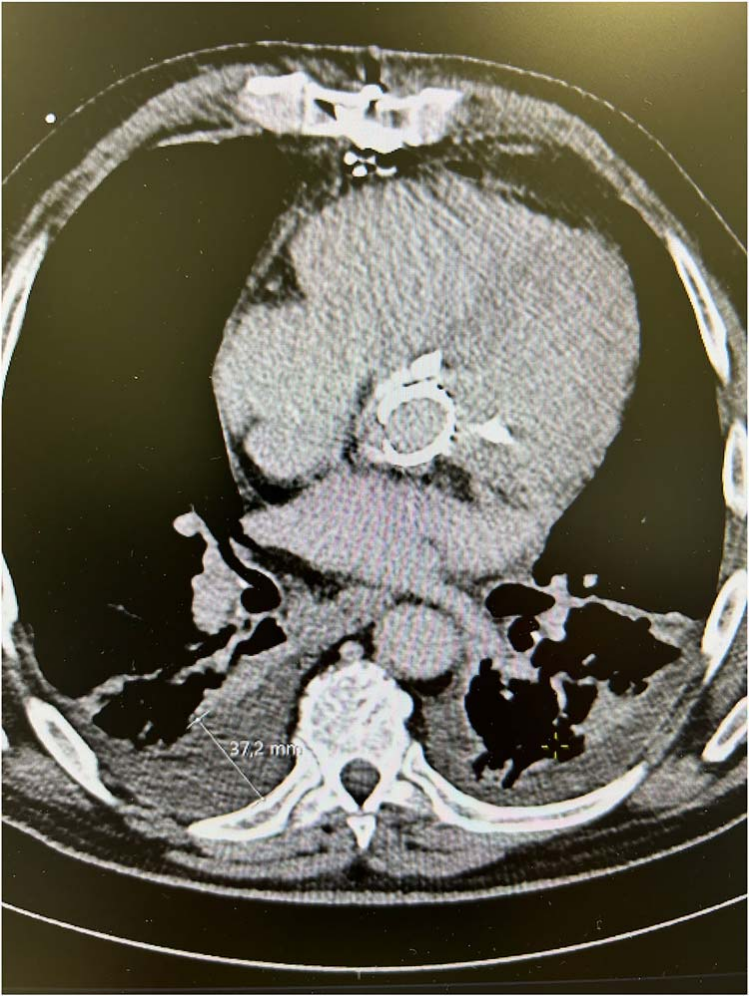
Preoperative computed tomography scans showing calcified aortic valve bioprosthesis in the aortic prosthesis together with pleural effusions.

**Figure 2:**
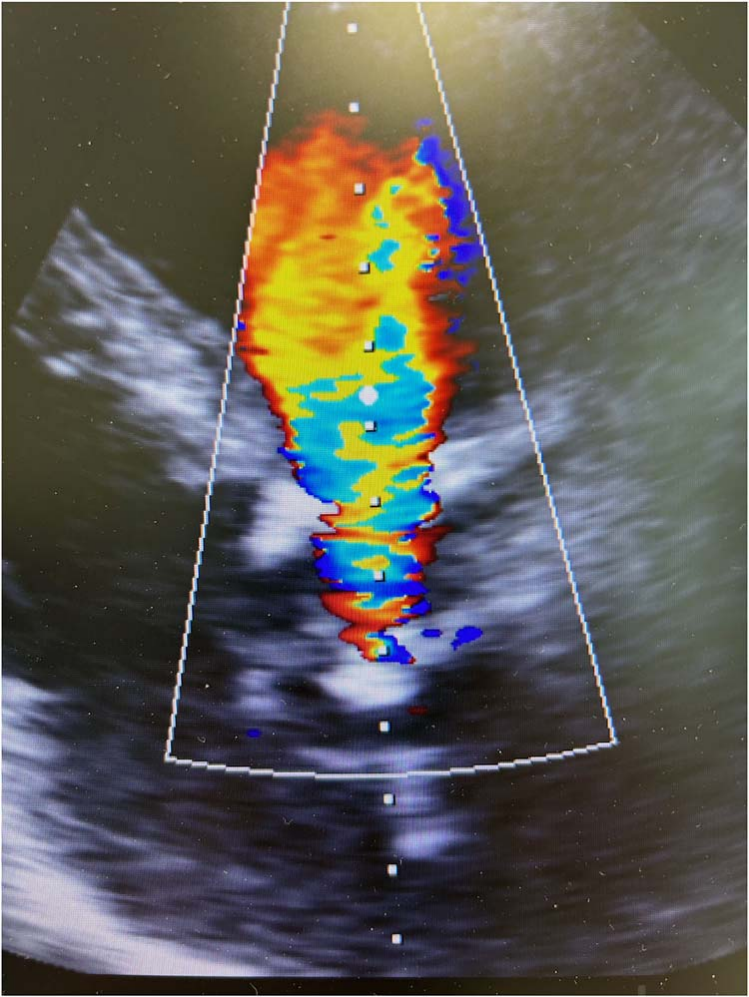
Preoperative transesophageal echocardiography showing significant regurgitation and stenosis of the aortic valve bioprosthesis.

The patient experienced significant dyspnea, increased passiveness, decreased performance, and cardiac echocardiography revealed a destroyed, regurgitant, and stenotic aortic valve prosthesis with myocardial calcification of the left outflow track (Fig. 1) including a peak mean aortic gradient of 76/49 mmHg, Vmax 4.3 m/s and AVA 1.0 cm^2^ and significant regurgitation (Fig. 2). Minimal mitral valve regurgitation and some coronary artery stenosis were also present.

A transcatheter aortic valve replacement (TAVR) solution was denied as calcification of the aortic valve prosthesis reached the coronary ostia that situated at the immediate vicinity of the annulus structure of the aortic valve prosthesis. The calcified spikes reached the intramyocardium of the left outflow track.

**Figure 3:**
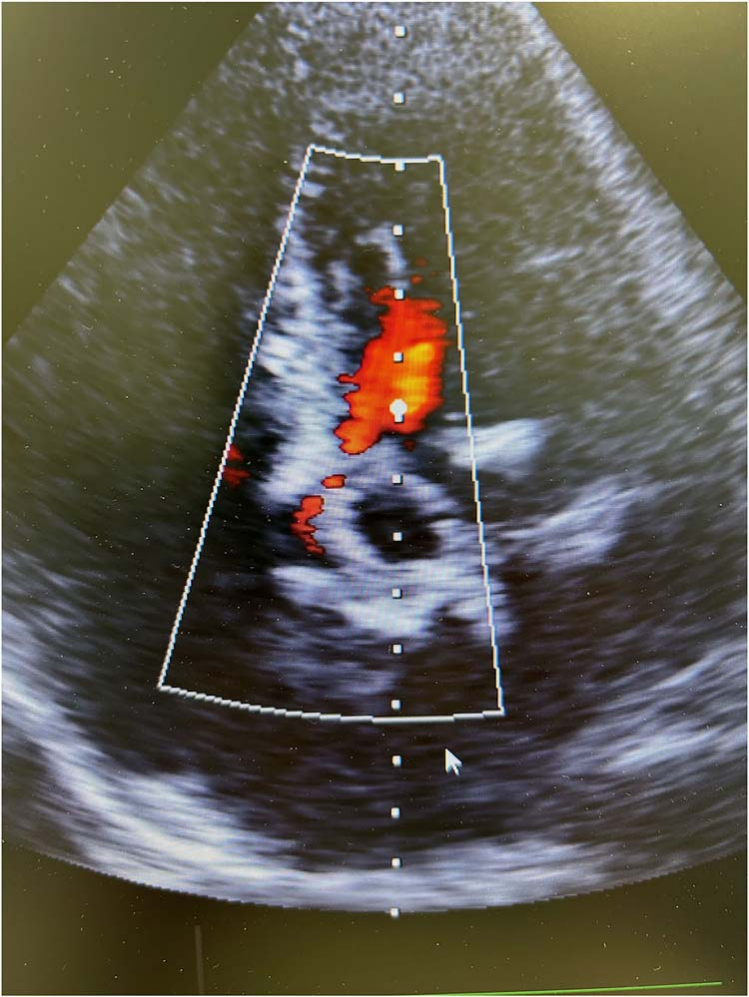
Postoperative transesophageal echocardiography showing nonsignificant regurgitation of a well-seated rapid-deployment Edwards Intuity bioprosthesis.

Resternotomy was undertaken and the aortic root was dissected free of considerable adhesions. The aortic arch and the remnant of the right atrial appendix were cannulated to initiate cardiopulmonary bypass and retrograde cardioplegia was administered after cross-clamping of the aortic prosthesis, which was thereafter transected immediately distally to the previous proximal aortic suture line. Care was applied while the destroyed aortic valve prosthesis and the coronary ostia were dissected free from the left outflow track encompassing the calcified annular structure, pledgets, and suture material. Though the aortic root remained intact after surgical revision, the remnant aortic valve annulus was torn and an intramyocardial defect was evident beneath the left and right coronary ostia. A size 23 mm rapid-deployment Edwards Intuity bioprosthesis was implanted with the aid of three guiding sutures that also ensured the integrity of the coronary ostia. The skirt of the prosthesis was raised up and the aortic valve prosthesis was well fit despite the revision lesions and intramyocardial calcium of the left outflow track beneath the old aortic valve annulus. After surgical closure of the supracoronary aortic prosthesis and weaning from cardiopulmonary bypass, echocardiography revealed a well-seated aortic valve prosthesis with a trace of paravalvular leak (Fig. 3). The patient experienced minimal weakness of the left arm during an otherwise uneventful recovery without cerebral lesions observed by computed tomography. Clopidrogel was readministered and the patient was dehospitalized after 10 days of surgery.

## Discussion

The rapid-deployment Edwards Intuity bioprosthesis was implanted after extracting the previously replaced stenotic aortic valve bioprosthesis together with major intramyocardial calcium deposits. The skirt of the bioprosthesis covered the annular and subannular lesions made during surgical revision without obstructing the stenotic coronary ostia.

Any aortic valve reoperation after implantation of an aortic valve bioprosthesis includes surgical risks such as tissue tear, emboli, and prolonged operation time. A valve-in-valve TAVR was not an option due to the relatively small annular and lumen sizes of the calcified aortic valve prosthesis, the pathological calcification endangering the coronary ostia, the atherosclerotic arterial access for a transcatheter bioprosthesis, and the structure of the left outflow track including the intramyocardial calcium deposits.

Surgical debridement of the destroyed aortic valve prosthesis and intramyocardial calcium through the previously implanted supracoronary aortic prosthesis, and implantation of a rapid-deployment aortic valve prosthesis ensured a feasible option by minimal manipulation of stenotic and calcified deposits, enlargement of the restricted left ventricle outflow track, securing the coronary ostia, and avoiding increased surgical duration without aortic root reconstruction despite the annular disruption after the revision process. Limitations include the need of follow-up using echocardiography as among all patients undergoing aortic valve surgery.
